# Movement and behavioral states of common carp (*Cyprinus carpio*) in response to a behavioral deterrent in a navigational lock

**DOI:** 10.1186/s40462-023-00396-z

**Published:** 2023-07-26

**Authors:** Maggie Raboin, John M. Plumb, Matthew D. Sholtis, David L. Smith, P. Ryan Jackson, Jose M. Rivera, Cory D. Suski, Aaron R. Cupp

**Affiliations:** 1 Contractor to U.S. Geological Survey, Upper Midwest Environmental Sciences Center, 2630 Fanta Reed Rd, La Crosse, WI 54603 USA; 2 U.S. Geological Survey, Columbia River Research Laboratory, 5501A Cook-Underwood Rd., Cook, WA 98605 USA; 3 Engineer Research and Development Center, U.S. Army Corps of Engineers, 3909 Halls Ferry Rd., Vicksburg, MS 39180 USA; 4 U.S. Geological Survey Central Midwest Water Science Center, 405 N Goodwin Ave, Urbana, IL 61801 USA; 5 U.S. Geological Survey, Upper Midwest Environmental Sciences Center, 2630 Fanta Reed Rd., La Crosse, WI 54603 USA; 6 Department of Natural Resources and Environmental Sciences, University of Illinois, 1102 S Goodwin Ave, Urbana, IL 61801 USA

**Keywords:** Invasive species, Hidden Markov models, Fish ecology, Acoustic telemetry, Control

## Abstract

**Background:**

Freshwater ecosystems are some of the most affected by biological invasions due, in part, to the introduction of invasive carp worldwide. Where carp have become established, management programs often seek to limit further range expansion into new areas by reducing their movement through interconnected rivers and waterways. Lock and dams are important locations for non-physical deterrents, such as carbon dioxide (CO_2_), to reduce unwanted fish passage without disrupting human use. The purpose of this study was to evaluate the behavioral responses of common carp (*Cyprinus carpio*) to non-physical deterrents within a navigation structure on the Fox River, Wisconsin. Acoustic telemetry combined with hidden Markov models (HMMs) was used to analyze variation in carp responses to treatments. Outcomes may inform CO_2_ effectiveness at preventing invasive carp movement through movement pinch-points.

**Methods:**

Carbon dioxide (CO_2_) was recently registered as a pesticide in the United States for use as a deterrent to invasive carp movement. As a part of a multi-component study to test a large-scale CO_2_ delivery system within a navigation lock, we characterized the influence of elevated CO_2_ and forced water circulation in the lock chamber on carp movements and behavior. Through time-to-event analyses, we described the responses of acoustic-tagged carp to experimental treatments including (1) CO_2_ injection in water with forced water circulation, (2) forced water circulation without CO_2_ and (3) no forced water circulation or CO_2_. We then used hidden Markov models (HMMs) to define fine-scale carp movement and evaluate the relationships between carp behavioral states and CO_2_ concentration, forced water circulation, and temperature.

**Results:**

Forced water circulation with and without CO_2_ injection were effective at expelling carp from the lock chamber relative to null treatments where no stimulus was applied. A portion of carp exposed to forced water circulation with CO_2_ transitioned from an exploratory to an encamped behavioral state with shorter step-lengths and a unimodal distribution in turning angles, resulting in some carp remaining in the lock chamber. Whereas carp exposed to forced water circulation only remained primarily in an exploratory behavioral state, resulting in all carp exiting the lock chamber.

**Conclusion:**

Our findings illustrate the potential of forced water circulation, alone, as a non-physical deterrent and the efficacy of CO_2_ injection with forced water circulation in expelling carp from a navigation lock. Results demonstrate how acoustic telemetry and HMMs in an experimental context can describe fish behavior and inform management strategies.

**Supplementary Information:**

The online version contains supplementary material available at 10.1186/s40462-023-00396-z.

## Background

Invasive species established in freshwater ecosystems can alter hydrological conditions and water quality, disrupt ecological communities, drive population declines and, in some cases, cause species extinctions [3, 21]. Globally, invasive carps (family *Cyprinidae*) are some of the most widespread freshwater invasives causing damage to ecosystems and economies alike [3, 6]. Where carps have already been introduced and established, like the Mississippi River in the United States, preventing further spread is a critical component of management plans [1, 7]. Recent approaches focus on placing behavioral deterrents at migratory pinch points along waterways, like lock and dams, to reduce the risk of invasive fish moving upstream without interfering with lock operation and navigation [33, 54, 55]. However, the effectiveness of non-physical deterrents may vary because not all fish respond similarly to a given stimulus [8, 15, 33, 53].

Fish within a population can vary widely in their responses to non-physical deterrents [8, 15, 33, 53]). For example, a wide range (3–40%) of the target population has been found to penetrate a given deterrent [8, 11, 29, 33, 53]. Variation in fish responses to deterrents can depend on size [34], social context [48], temperature [47], and stress level [49]. Attention is typically paid to individuals that exhibit a predicted behavioral response to a given stimulus but understanding those individuals of a population that do not respond to deterrents may be key to making informed decisions about deterrent effectiveness and implementation strategies [43].

Acoustic telemetry combined with hidden Markov models (HMMs) offer the ability to collect fine-scale movement data and explore behavioral variation of individuals [27, 30]. An animal’s movement path is composed of several behavioral states that depend, in part, on the timescale of interest [32]. For example, an animal might transition between states related to foraging or resting across days and exploratory or encamped states across minutes. For fish, these behavioral states are often visually unobservable but can be inferred from a combination of measures from their acoustic trajectories (e.g., step-length, turning angles, acceleration) [27, 28]. HMMs are a class of statistical models that can detect states and state changes in time series data like that collected by acoustic telemetry [27, 30]. When applied in an experimental or invasion context, HMMs can provide essential information about variation in fish behavior and state transitions in response to experimental deterrents and management applications.

Carbon dioxide (CO_2_) is a recently approved behavioral deterrent to limit invasive carp movement [50]. Fish exposed to elevated CO_2_ in freshwater often exhibit a strong avoidance response or may become incapacitated at more extreme concentrations and exposure durations [8–11, 16, 25, 47]. Managers could use these behavioral responses to potentially deter movements or expel fish from areas where they are at risk to move upstream, such as movement pinch-points at lock and dam structures. However, until recently, the scalability and efficacy of using CO_2_ as a deterrent in large-scale and real-world scenarios had yet to be demonstrated. In 2019, the first large-scale delivery system for CO_2_ was designed, constructed, and operated for research purposes at a navigation lock structure on the Fox River in Kaukauna, Wisconsin, USA. This project aimed to evaluate feasibility and outcomes of a CO_2_ injection system built to expel carp from a lock, thereby limiting carp movement upstream during lock operation [55]. The pump injection system design required kinetic energy and forced water circulation from a series of jets to mix CO_2_ throughout the water column of the lock [54, 55].

The purpose of our study is to characterize the influence of CO_2_ applications on movement and behavioral states of invasive carp at a lock on the Fox River near Kaukauna, Wisconsin, USA. We first measured common carp (*Cyprinus carpio*) responses to CO_2_ injection with forced water circulation into the chamber water, forced water circulation of water without CO_2_, and a null scenario without CO_2_ injection or forced water circulation. We then used HMMs to distinguish and define fine-scale carp behavioral states and evaluate the effect of treatment applications. We predicted that more carp would leave the lock in response to CO_2_ injection with forced water circulation than under forced water circulation without CO_2_ or the null treatment. We expected that behavioral states would align with this prediction and show carp maintaining or transitioning to an “exploratory” state (longer step length, broader turning angle distribution) resulting in a more consistent exit time from the lock in response to CO_2_ injection with forced water circulation, whereas behavioral states would be more mixed in the presence of forced water circulation without CO_2_ or under the null treatment. Our results are important for understanding the effectiveness of CO_2_ as a large-scale management tool to prevent the spread of invasive carp and will have broad implications for understanding behavioral variation of invasive species populations in response to management strategies.

## Methods

### Study location

**Fig. 1:**
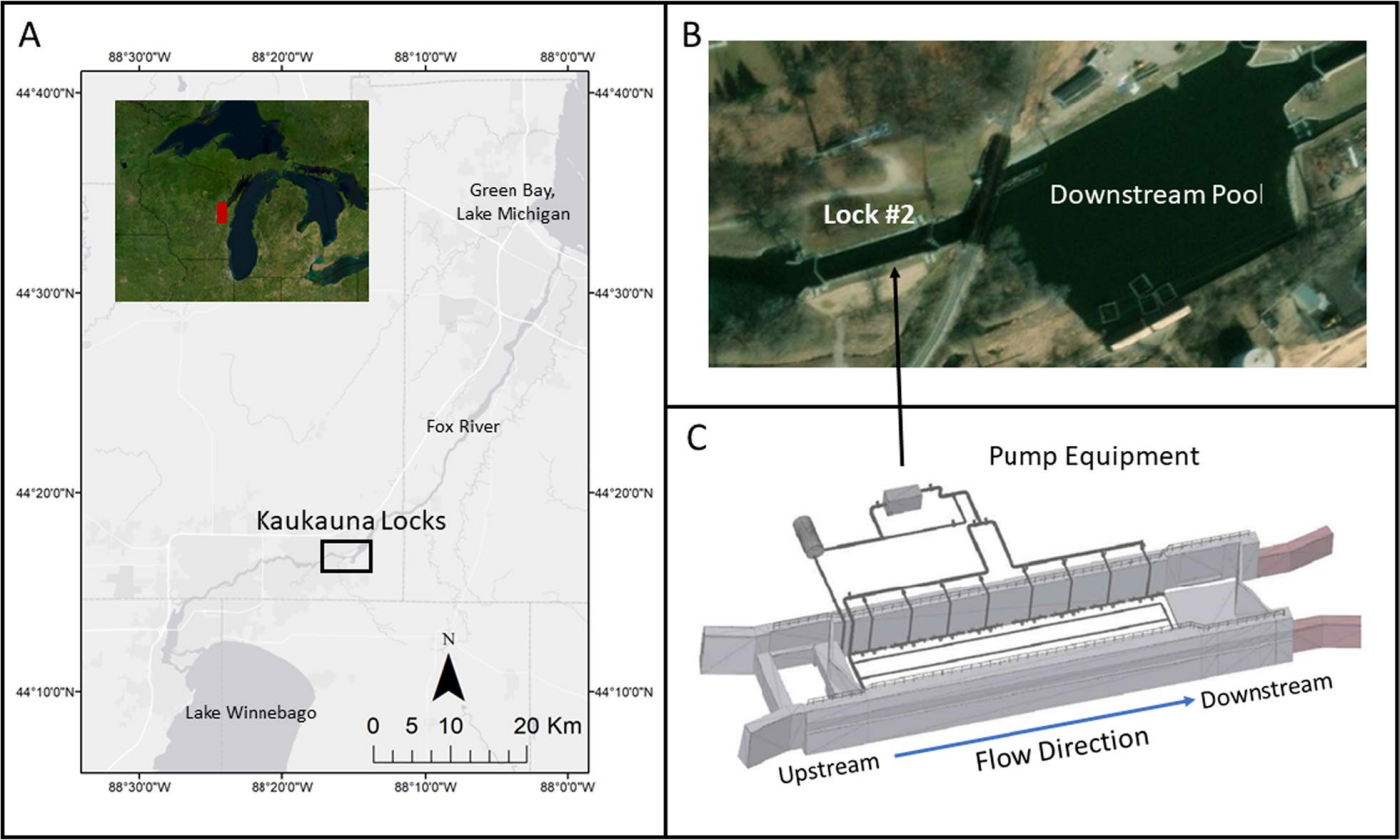
**A** Research and equipment location in Kaukauna, Wisconsin, USA. **B** Aerial image of Lock #2 and downstream pool (base map from Esri and its licensors, copyright 2022). **C** CO_2_ injection system including pump equipment. Detailed information on the CO_2_ delivery system is available in Zolper et al. [55]

The Kaukauna locks are a series of five navigation locks on the Fox River near Kaukauna, Wisconsin, USA (Fig. 1). Locks are operated by the Fox River Navigation System Authority and were offline to navigation during this study with restricted public access. The CO_2_ injection system was constructed and operated on the north bank adjacent to Lock 2 with carp monitoring using acoustic telemetry receivers throughout the lock chamber and immediate downstream pool (Fig. 1). Lock dimensions were 51.82 m (m) long and 11.12 m wide at the top, tapering down to 10.72 m wide at the bottom with a volume of about 2,920,000 ± 47,000 L at head-water level (5.08 m).

### Water quality

Water-quality data were measured continuously throughout the lock chamber using small, tethered boats equipped with water-quality sensors and radio telemetry. Five taglines were installed across Lock 2 at roughly equal intervals. A boat was suspended from each tagline on a pully system so the boat could be moved across the lock during the trials. Two multi-parameter sondes, at ~ 0.3 m and ~ 1.5 m below water surface, with water temperature (degrees Celsius [°C]), pH (-log scale from 1 to 14), and dissolved oxygen (milligrams per liter [mg/L]) sensors, were suspended from each boat on a weighted line.

### Study fish and tagging

Common carp (*Cyprinus carpio*) were collected from the Fox River downstream from the study location using common electrofishing techniques. Following collection by electrofishing crews, carp were transported to the test site using hauling trucks and transferred into large holding pens in the Fox River until the time of testing. The carp species used in this study, common carp, was chosen due to their similarity in size to bigheaded carps (*Hypophthalmichthys* spp.) (500+ millimeters [mm]) and their abundance in the area. There was no mechanism to bring bigheaded carp to the study site, therefore common carp were the only reasonable proxy for invasive carp in this study. Common carp ranged from 409.6 to 788.9 mm total length (mean = 621.6; standard deviation [SD] = 73.74), and, using the equations provided by Tessema et al. [46], weighed between 900 and 6150 g (g). Animals were collected and transferred under a Wisconsin Department of Natural Resources NR40 permit with handling and experimental procedures approved by U.S. Geological Survey (USGS) Animal Care and Use Committee protocol AEH-19-CO2LOCK-01.

The carp were removed from the net pens and outfitted with external acoustic transmitters [40]. Each carp was externally outfitted with a transmitter (model 795LD, Hydroacoustic Technology Incorporated HTI, Seattle, Washington) that was bonded to a T-bar tag (Floy Tag and Mfg, Seattle, Washington) by the manufacturer. Transmitter pulse rates were set to short intervals and ranged from 1301 to 5862 ms (ms) (1.3–5.9 s [s]) to minimize gaps between position estimates. Each transmitter was 6.8 mm in diameter, 20 mm long and weighed 1.0 g in air. Carp were sedated with 100 mg/L AQUI-S 20E (AQUI-S New Zealand, Lower Hutt, New Zealand) to facilitate handling before external transmitter attachment [9, 10]. Transmitters were attached to carp using the T-bar tag placed approximately 1 cm (cm) lateral and posterior to the midline of the dorsal fin. After transmitters were attached, three common carp were stocked directly into the lock chamber for acclimation overnight (acclimation times were typically 16–20 h [h]) and CO_2_ treatments were initiated the next day. This created several independent replicates across the established treatment groups. Data from carp that were previously tagged and released were also collected, but observations were limited and opportunistic depending on the starting locations of those individuals when a given treatment was initiated.

### Acoustic telemetry detection system

The study area was equipped with two independent, 16-hydrophone acoustic telemetry receivers (Model 290; Hydroacoustic Technology Inc. [HTI], Seattle, USA) attached to Windows OS based collection computers utilizing vendor supplied software AcousticTag for raw data acquisition. Each receiver had an attached GPS antenna for time synchronization data stored in the raw files allowing time alignment of the independent receivers. Eight hydrophones were deployed in the lock with four more downstream from the lower lock gates contributing to position solutions of carp in the lock chamber. Hydrophones were mounted vertically to metal plate mounts held in place by 190 kg (kilograms) of sandbags. Each hydrophone location was recorded using a Trimble R10 receiver collecting in a real-time kinematic global positioning system (RTK-GPS) with corrections provided through a virtual reference station service (horizontal accuracy 8 mm + 1 parts per million [ppm] root mean square [RMS]).

Raw acoustic transmitter echoes recorded in AcousticTag must be post processed for identification and selection from background acoustic noise signals in the environment due to the nature of pulse rate transmitters compared to phase shift key encoding utilized by other systems [18]. MarkTags is a vendor supplied software application allowing users to view, select, and output transmitter signal records on individual hydrophones for further interrogation. These records are then imported into AcousticTag to perform positioning estimate calculations using mean squared difference of arrival time from four or more hydrophones [17]. The USGS developed a custom application to perform these tasks to create a mostly automated and scalable data processing routine with greater flexibility in adjusting parameters to best fit specific field site conditions. This application uses a histogram driven search with convolution marking and heuristic filtering to identify and separate transmitter echoes from other acoustic signals in the raw data. Files created by application were then processed to generate position estimates using a genetic algorithm differing from the multilateralization approach in AcousticTag. USGS selected the use of these custom solutions after finding comparable output performance, site specific optimization flexibility, and gains in processing time and cost efficiencies [5]. Measuring the performance of the acoustic detection system is difficult and imperfect because two measurement systems (sources of error) must be considered: (1) the RTK-GPS system and (2) the hydroacoustic telemetry system. Given this, accuracy of each acoustic receiver was measured using nine test tags with a pulse rate between 2032 and 6495 ms (2.0–6.5 s) maneuvered around the lock chamber attached to a small inflatable vessel. The vessel was fitted with an RTK-GPS set to record a position every second, mounted to a vertically orientated pole directly above the tags. The test tag positions were estimated using a time difference of arrival positioning algorithm and merged by time to the nearest RTK-GPS position, with a tolerance less than or equal to one second. The distance between the test tags and RTK-GPS positions was calculated in the x and y dimensions with a median difference of 0.878 m (SD = 1.22). Given this, we assume positioning error was sufficient in positioning carp at various locations throughout the lock. We did not test positioning accuracy under the various test conditions and assumed similar positioning error across treatments and trials.

### Treatments

CO_2_ was injected into the lock chamber using several different system configurations to evaluate engineering system performance [55]. Due to the scale of CO_2_ application in this study, additional engineering considerations were made to ensure rapid and uniform mixing of CO_2_ in the lock chamber that were not addressed in previous studies [55]. This system used kinetic energy and forced water circulation from a series of jets to create a semi-recirculating loop that increased the concentration of CO_2_ in the water column of the lock chamber over time. Pump volume flow rates of 6810–12,110 L per minute (L/min) and gas volume flow rates of 1134–1588 kg per hour (kg/h) were necessary with this gas injection system to reach target concentrations of 100–150 mg/L CO_2_ (i.e., the prescribed treatment concentrations on the pesticide label) within 5–10 min [55]. Concentrations of ~ 100 mg/L have previously been shown to induce avoidance behavior in both laboratory and pond settings [16]. Detailed information on the CO_2_ delivery system, operating parameters, and lock operation are reported in Zolper et al. [55].

Treatment groups for carp behavior assessments were determined based on how the injection system was operated. We measured the response of tagged carp in relation to CO_2_ concentration, water temperature, and forced water circulation over 20 experimental trials (Table 1). Trials were conducted using (1) CO_2_ injection with forced water circulation, (2) forced water circulation only but no CO_2_ injection and (3) null = no forced water circulation or CO_2_ injection. Because forced water circulation is required to facilitate CO_2_ mixing in water throughout the lock chamber, we did not inject CO_2_ without forced water circulation.

**Table 1:** Summary of the number tagged carp and the total number of locations by experimental trial and treatment

Trial	Treatment	Number of carp	Total observations
1	CO_2_	3	2048
3	CO_2_	3	6669
5	On	3	8084
7	On	3	10,333
8	CO_2_	3	16,044
9	CO_2_	2	13,531
11	On	3	12,297
13	CO_2_	3	14,122
15	Off	3	13,922
16	CO_2_	3	14,413
18	On	2	14,733
20	On	3	17,166
22	CO_2_	3	18,567
23	CO_2_	3	21,485
25	On	3	15,139
27	CO_2_	3	15,115
29	Off	3	14,889
30	CO_2_	3	13,743
32	CO_2_	3	15,958
34	CO_2_	2	20,461

A typical trial began when the lock chamber was emptied to tailwater level with the downstream gates closed. Carp were then tagged and placed into the closed lock chamber for an overnight acclimation period. The next day, baseline water quality was measured throughout chamber and instruments returned to the centerline of the lock chamber. Pumps then started and circulated water within the lock chamber. CO_2_ was then injected into the recirculated water (for applicable trials where CO_2_ was tested) and the downstream gates were opened to provide carp an opportunity to vacate the lock chamber. Injection continued until target CO_2_ concentration were reached. After reaching target concentration, the pumps were turned off and telemetry data with water quality monitoring was continued for approximately 1 h. At the conclusion of each trial, the lock gates were closed, and the lock was flushed until water quality returned to baseline or ambient conditions. Experiments took place between August 5 and September 6, 2019.

### Statistical approach and methods

We quantified the ability of CO_2_ injected into a lock chamber with forced water circulation to expel carp from the lock and act as a deterrent to carp movement by testing the effectiveness of CO_2_ concentration, forced water circulation, and temperature on carp movement through two quantitative analyses—a descriptive data summary and HMM. We first summarized the overall response of the carp to the experimental treatments using Kaplan–Meier estimates to examine the time-to-exit the lock when exposed to the three experimental treatments. Time-to-event analyses, in this case time-to-exit, have been shown to be effective at assessing fish passage at hydroelectric dams [4].

We used an HMM to define the behavioral responses that gave rise to our time-to-exit results and inform deterrent implementation. The HMM allowed us to classify carp movement and detection locations into latent behavioral states and was informed using carp step length (distance traveled per unit time) and turning angle (change in direction from time *t* to time *t* + 1). We followed the methods of McClintock and Michelot [31] and estimated the parameters of the HMM using the ‘momentuHMM’ package in R software [39]. To provide the modeling framework with a regular time series of carp locations we first fit a correlated random walk (CRW) model using the ‘crawl’ R package [24, 31, 39]. The CRW estimated carp locations (latitude and longitude) every 6 s (s) from the irregular time series carp locations recorded from transmitters. We chose a 6 s time step because enough observed data were available to provide robust estimates of a carp’s position at a relatively short time scale.

Our CRW model was then used to fit a two-state HMM. We chose two states based on the timescale of our study and its biological relevance. For each carp we collected data for up to 90 min or until the carp exited the lock. Therefore, complex models to estimate more than two latent states for the carp used in this study was not warranted [36].

The HMM models the transition probabilities between the behavioral states as well as the step length and turning angle distribution parameters. Step lengths were assumed to follow a gamma distribution and turning angles were modeled using a von Mises distribution [31]. Both the state transition probabilities and the mean step length were modeled as a function of forced water circulation, CO_2_ concentration (mg/L) and water temperature (°C). We did not include experimental effects on turning angle, and so only the mean (and SD) of the turning angle distribution was estimated. We included the effects of trial (*R*) accounting for 20 levels, forced water circulation (*P*) as a factor having two levels (off or on) and CO_2_ concentration and mean water temperature were included as continuous covariates. For example, transition probabilities, $\psi_{i,j}$, between the two behavioral states (*i* and* j*; state 1 → 2 or state 2 → 1) may be expressed as a linear function of covariate effects:

1 $$\psi_{i \to j} = \beta_{{0_{i \to j} }} + \beta_{2} R_{i \to j} + \beta_{3} P_{i \to j} + \beta_{4} CO_{2}{i \to j} + \beta_{5} T_{i \to j}$$

where $\beta_{0 - 5}$ are the coefficients for the intercept, trial, forced water circulation, CO_2_ concentrations, and water temperature (*T*), respectively. In a similar manner, coefficients ($\theta$) on the mean step length ($\lambda_{k}$) given behavioral state (k) were modeled as a linear function of the same covariates:

2 $$\lambda_{k} = \theta_{0k} + \theta_{1} R_{k} + \theta_{2} {P_{k}} + \theta_{3} CO_{2k} + \theta_{4} T_{k} .$$

We assessed model fit by examining bivariate and quantile plots of the pseudo residuals (see Additional file 1). Lastly, we explore, in graphic and tabular form, the estimated probabilities of remaining in a behavioral state as well as the effect of the experimental conditions on the mean step lengths and turning angles of the carp.

## Results

### Time-to-exit analysis

**Fig. 2:**
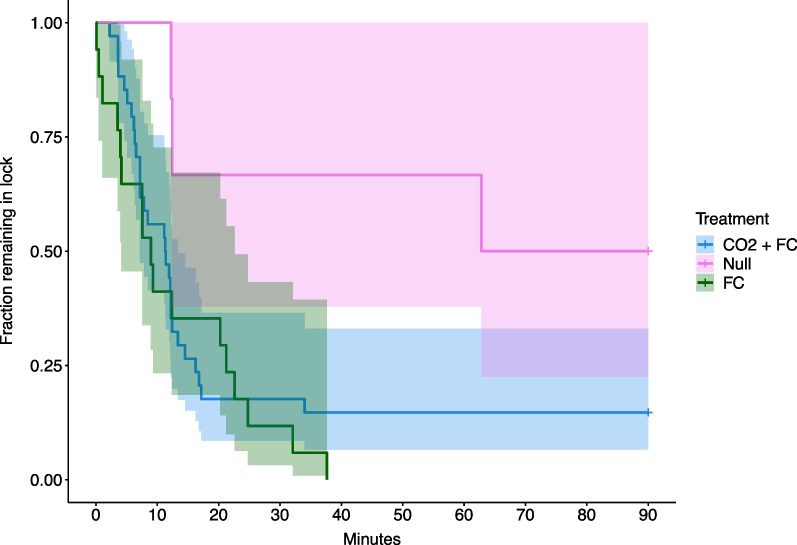
Kaplan–Meier curves of exit times by acoustic-tagged common carp exposed to three experimental treatments, including CO_2_ with forced water circulation (CO_2_ + FC), null, and forced water circulation only (FC), at the Kaukauna Locks, Wisconsin, USA

Common carp in our study had a shorter and more consistent response in their time to exit the lock when exposed to CO_2_ with forced water circulation and forced water circulation only, than those in the null treatment. Setting the exit times of the carp that did not leave the lock to the time horizon of the trials resulted in median exit time of 8.9 min during forced water circulation only, 11.3 min with CO_2_ with forced water circulation, and 76.4 min when no stimuli was applied. The fraction of tagged carp that remained in the lock between trials and experimental treatments indicated that the forced water circulation only and CO_2_ with forced water circulation treatments were successful at eliciting a response from the carp, including exit from the lock (Fig. 2). Out of 34 carp at the start of the CO_2_ with forced water circulation treatment, 5 carp (proportion [p] = 14.7%; standard error [SE] = 6.07%) remained in the lock for the duration of the trial. Of the 6 carp at the start of the forced water circulation off treatment, 3 carp (p = 50%, SE = 2.04%) remained in the lock at the end of the trial and all 17 carp at the start of the forced water circulation on (no CO_2_) treatment exited the lock before the end of the trial.

### Behavioral states and transitions

**Fig. 3:**
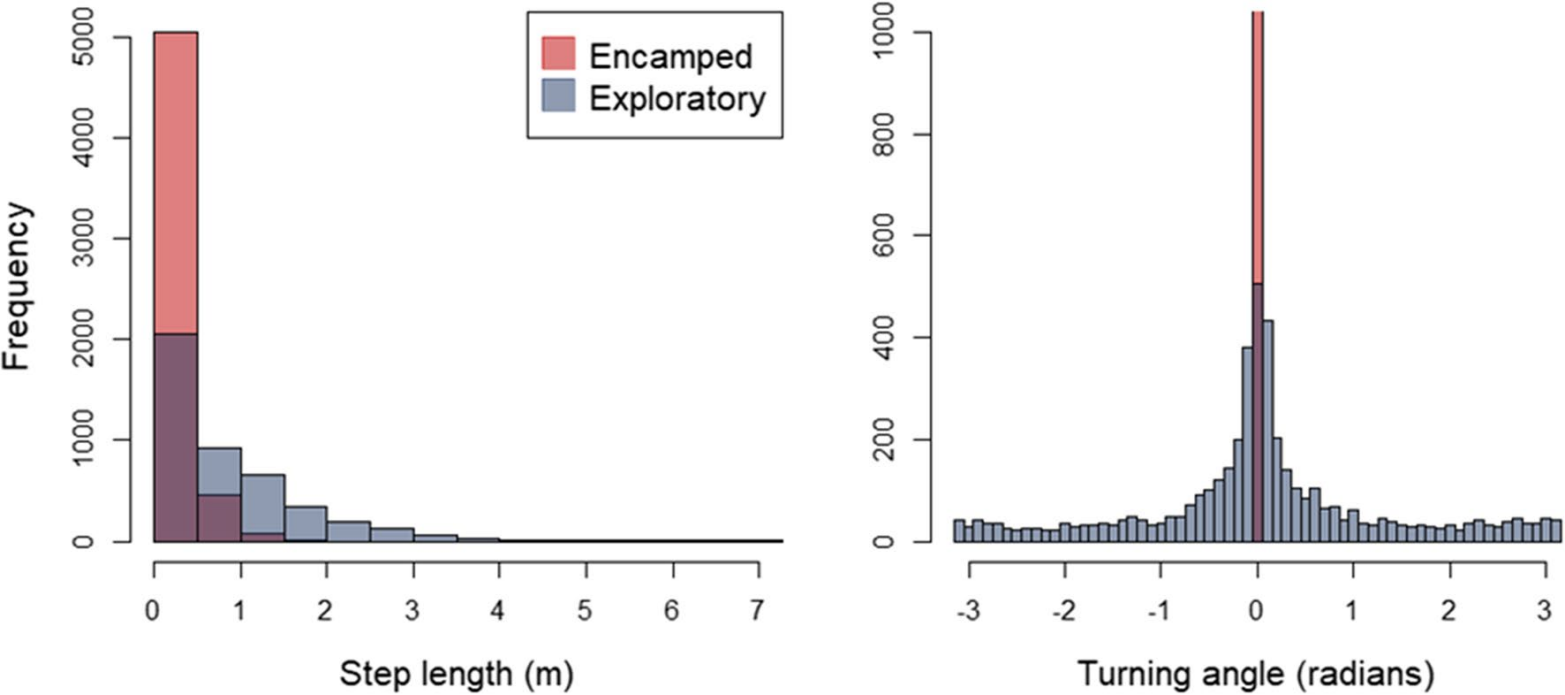
Distribution of step lengths and turning angles by the modeled behavioral states

Our HMM was able to identify and characterize the step lengths and turning angles of the tagged carp into one of two behavioral states (Fig. 3). The exploratory state was characterized by a long step length, with carp moving a mean of 0.988 m (SD = 0.928) every 6 s, and a lack of concentrated turning angles (mean = − 3.1414 radians; concentration = 0.00002) indicating frequent change of direction. In contrast, the encamped state was characterized by a short step length of 0.026 m (SD = 0.019) every 6 s and more concentrated turning angles (mean = − 0.00008 radians; concentration = 0.0009), indicating movement direction was often similar (correlated) among successive positions. However, the mean of the turning angle distribution was near zero during both the exploratory and encamped states.

**Fig. 4:**
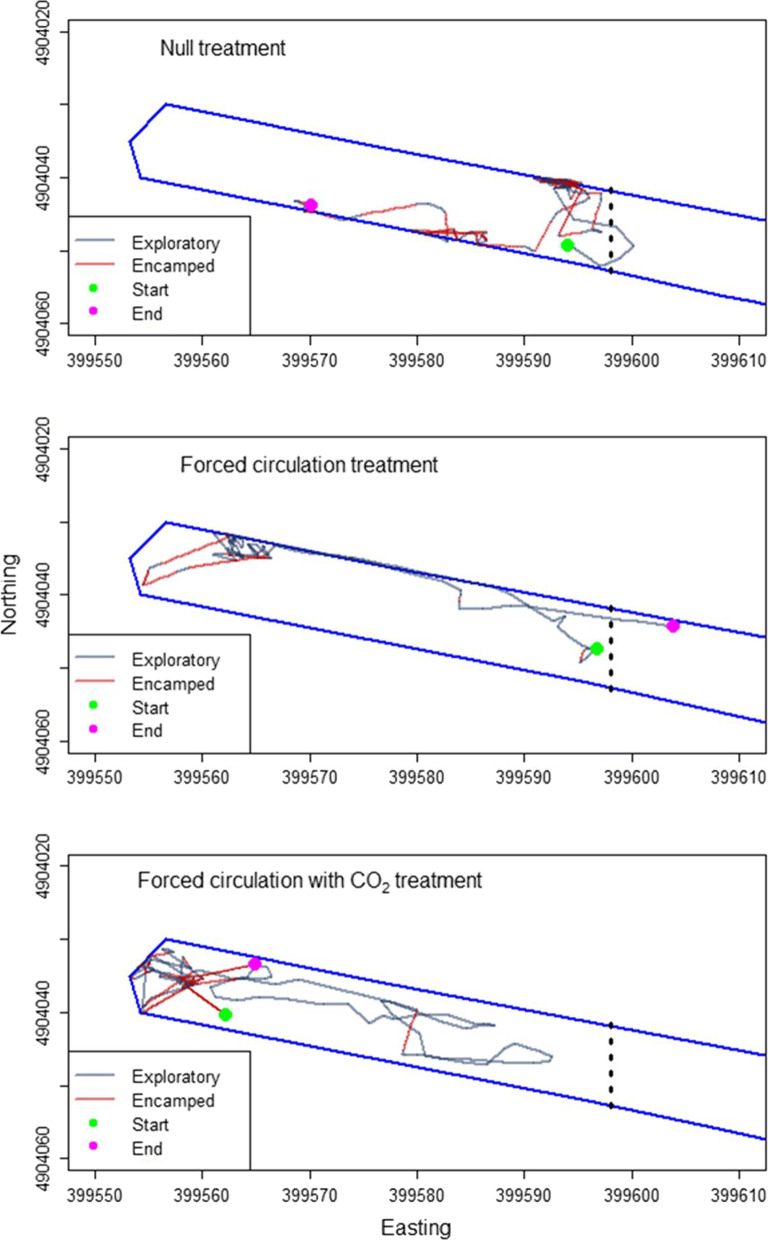
Example path for an individual carp in each treatment, colored by behavioral state. Dashed vertical line represents the open gate of the navigational lock

Assessment of HMM fit indicated the assumed underlying distributions were reasonable. Quantiles of the theoretical (model) and sample (data) residuals for step lengths and turning angles were reasonably well matched, and pseudo residuals in step length and turning angles appeared bivariate normal in distribution (see Additional file 1). Our assumptions about the underlying distributions in step length (gamma) and turning angles (Von Mises) appeared to be reasonable (Fig. 4).

**Fig. 5:**
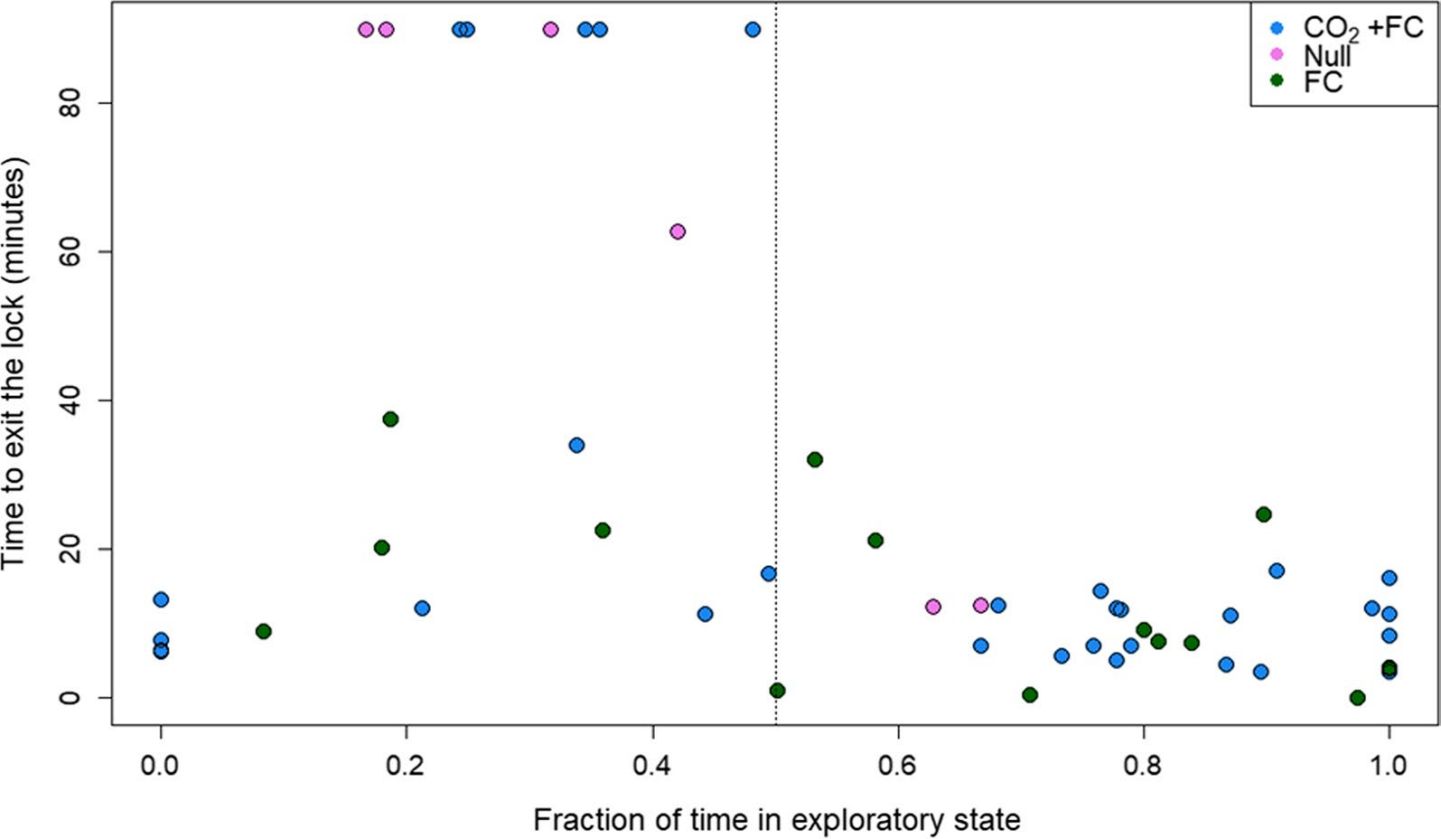
Relationship between the fraction of time acoustic-tagged common carp were in the exploratory state and the time for the carp to exit the lock during three experimental treatments, including CO_2_ with forced water circulation (CO_2_ + FC), null, and forced water circulation only (FC). The dashed line designates the point when carp spent 50% or more of their time in the exploratory state. Carp that did not exit the lock by the end of the trail were assigned an exit time of 90 min

The overall fraction of time that carp were in the exploratory behavioral state was 44.5% (4,508 of 10,125 six-second locations). Individual variation in the fraction of time spent in the exploratory behavioral state ranged from 0 to 100% (Fig. 5). The fraction of time spent in the exploratory state in relation to the exit times of the carp indicated a visual threshold at about 0.5, where carp spent more than 50% of their time in the exploratory state. Above this threshold, all carp exited the lock in less than 32 min. Below this threshold, when carp spent most of their time in the encamped state, carp exit times from the lock were longer, more variable, and included all individuals that did not exit the lock over the trial duration. Thus, factors that increase an exploratory state in carp would likely lead to carp finding the open downstream gate and exiting the lock consistently.

**Fig. 6:**
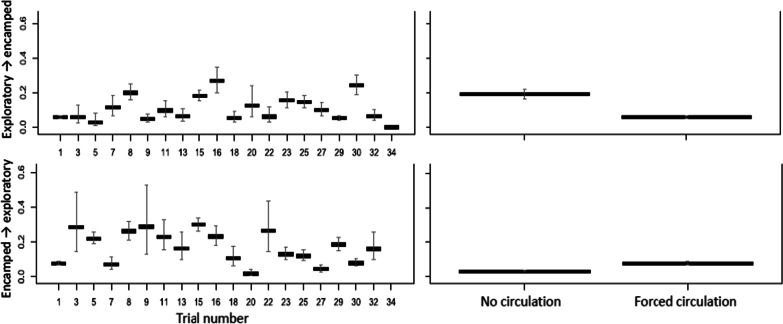
The effect of trial and forced water circulation on the probability of transitioning from the exploratory to encamped behavioral state (top panels) and from the encamped to exploratory behavioral state (bottom panels). Bars represent the 95% confidence intervals about the state transition probabilities. Note values are presented at the reference categories of the factors and the mean of the modeled covariates

The effect of covariates on the probability of carp transitioning to an exploratory state support the findings from the time-to-exit analysis (Table 2). The HMM, however, used more of the available data and provides more detail about how the experimental effects gave rise to the observed differences in carp exit times. Variation among trials was not trivial, and accounting for trial-to-trial differences was necessary to better measure the experimental effects of interest (Fig. 6). Forced water circulation had a positive effect on the transition probability to an exploratory behavioral state (Fig. 6). Given a carp was in an exploratory state, forced water circulation reduced the probability of the carp transitioning to an encamped state by about half. If a carp was in the encamped state, forced water circulation had a more modest effect on the probability of transitioning to an exploratory state. Carp in the encamped state appeared less responsive to forced water circulation than carp in the exploratory state.

**Table 2:** Coefficients for the experimental effects on the transition probabilities and mean step lengths of acoustic tagged carp for each trial

Parameter	Transition probability		Mean step coefficient	
	Exploratory→Encamped	Encamped→Exploratory	Exploratory	Encamped
Intercept	− 2.2094	1.7924	− 1.6223	0.1986
Trial 3	− 0.0022	1.5630	− 0.1591	0.6082
Trial 5	− 0.7357	1.2176	0.1972	1.1703
Trial 7	0.7245	− 0.1150	− 0.1162	− 0.2302
Trial 8	1.3874	1.4498	− 0.2938	− 0.0974
Trial 9	− 0.2170	1.5778	0.0819	0.9923
Trial 11	0.5541	1.2692	0.1232	0.4183
Trial 13	0.0836	0.8403	− 0.1762	0.0872
Trial 15	1.2704	1.6300	− 0.2436	− 0.0655
Trial 16	1.7770	1.2854	− 0.4697	− 0.5593
Trial 18	− 0.0840	0.3666	0.0488	− 0.1504
Trial 20	0.8212	− 1.5633	− 0.1033	− 0.5408
Trial 22	0.0584	1.4563	0.1803	0.8814
Trial 23	1.0770	0.5765	− 0.1256	− 0.5767
Trial 25	1.0040	0.4858	− 0.2043	− 0.6236
Trial 27	0.5568	− 0.6091	0.1227	− 0.3080
Trial 29	− 0.0950	1.0060	0.1948	− 0.0547
Trial 30	1.6406	0.0208	− 0.4584	− 0.8583
Trial 32	0.0796	0.8260	0.3547	0.2255
Trial 34	− 7.0871	11.5272	− 0.2096	− 0.2432
Pump—off	− 1.3368	0.9893	0.1478	0.4132
CO_2_ (mg·L^−1^)	0.0096	0.0010	− 0.0014	− 0.0015
Temperature (°C)	0.0223	− 0.2335	0.0662	− 0.0847
Std. deviation			− 0.07454863	− 1.665357
Zero mass			− 8.450786	− 15.27187

**Fig. 7:**
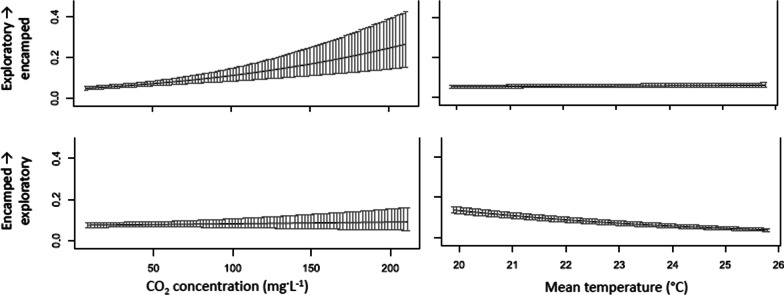
The effect of trial and pump operation on the probability of transitioning from the exploratory to encamped behavioral state (top panels) and from the encamped to exploratory behavioral state (bottom panels). Bars represent the 95% confidence intervals about the state transition probabilities. Note values are presented at the reference categories of the factors and the mean of the modeled covariates

Higher CO_2_ concentrations increased the probability of carp transitioning from an exploratory to an encamped behavioral state and decreased the probability of carp transitioning from an encamped to an exploratory state, thereby decreasing carp activity and movement within the lock (Fig. 7). The mean temperature of the trial appeared to have little effect on the state transitions of the carp, but we did measure a decrease in the transition probability from encamped to exploratory behavioral states as temperature increased (Fig. 7).

### Step lengths given the behavioral state

**Fig. 8:**
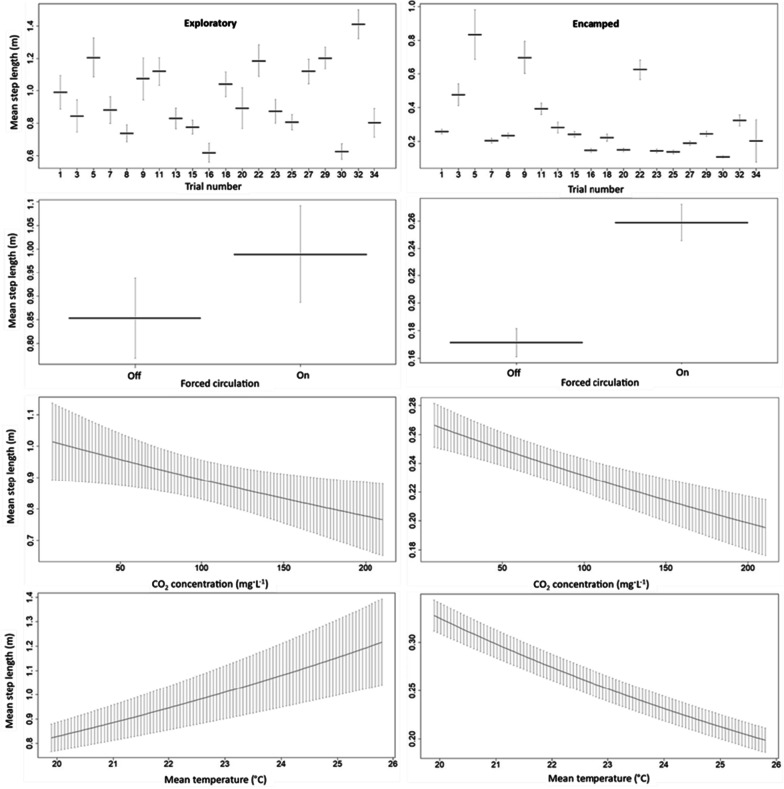
The effect of trial, forced water circulation, CO_2_ concentration, and mean water temperature on the mean step lengths of acoustic-tagged carp given an exploratory or encamped behavioral state. Bars represent the 95% confidence intervals of mean step length. Note values are presented at the reference categories of the factors and the mean of the modeled covariates

Given a behavioral state, coefficients for the experimental effects on the mean step lengths of the tagged carp are provided (Table 2). The modeled covariates had a stronger measured effect on the step length distribution during the exploratory state. The covariates had a relatively smaller effect on carp step lengths when in the encamped state. This observation is supported by comparing the range in mean step length over range of the covariate between the behavioral states (Fig. 8). The effects of forced water circulation and CO_2_ had a similar directional effect among the behavioral states with forced water circulation increasing the mean step length and higher CO_2_ concentrations decreasing the mean step length. The directional effect of temperature on mean step length was opposite between the behavioral states such that carp in the exploratory state had longer mean step lengths at higher temperatures and carp in the encamped behavioral state had slightly shorter mean step lengths at higher temperatures.

## Discussion

We found forced water circulation—with and without CO_2_—effective at expelling carp in situ from a large-scale navigation lock. Contrary to our predictions, forced water circulation alone resulted in faster exit times for carp and more carp exiting the lock over time than CO_2_ with forced water circulation. Hidden Markov modelling revealed that this finding was due to individual variation in carp responses to CO_2_ injection with forced water circulation. Under greater concentrations of CO_2_, carp were more likely to transition to an encamped state and have shorter mean step lengths, resulting in slower exit times and fewer carp leaving the lock over time. Our findings indicate that forced water circulation alone may be an effective non-physical deterrent to carp movement and that combing descriptive measures with HMMs could help inform implementation strategies of non-physical deterrents, including the use of CO_2_ in other contexts and deterrent combinations.

The system design in this study required turbulent mixing to distribute CO_2_ throughout the lock chamber and was present in the forced water circulation—with and without CO_2_—treatments [55]. Following an acclimation period, carp were exposed to a sudden and abrupt increase in water circulation throughout the lock chamber, which likely was a substantial disruption to carp. Turbulence is not often considered in the collection of potential non-physical deterrents to movement of invasive fish and therefore was not the focus of our study. Still, our results from the forced water circulation without CO_2_ treatment and other evidence indicate it might be effective in this context. For example, turbulence can influence swimming biomechanics and fish passage through fishways [19, 42, 44]. It is also known to cause disorientation and induce avoidance behaviors [19, 37, 42, 45] and has been shown to increase mortality in early life stage invasive carp [38]. Our results indicate that forced water circulation increased carp step-lengths, resulting in consistent exit from the lock. Future research might seek to understand the relationship of different turbulence metrics, including rate-of-change, in relation to carp avoidance thresholds, turbulence as a barrier to carp passage, and whether carp may habituate to turbulence overtime. It could be that the rapid onset of turbulence in the lock chamber is more effective at eliciting a response than a more gradual introduction. Additionally, future engineered CO_2_ injection systems, such as the forced water circulation pump system tested in this study, may consider intentionally incorporating turbulence to enhance the behavioral response of carp to deterrents.

Variation in carp responses to increasing CO_2_ concentrations might be attributed to differences in CO_2_ tolerance across individuals or differential stress response strategies. In our study, a proportion of carp responded to increasing CO_2_ concentrations (a maximum of 150 mg/L for 30 min) by transitioning to an encamped state. Previous studies reported fish succumbing to the anesthetic properties of CO_2_ and/or losing equilibrium within the range of CO_2_ concentration relevant to our study but for periods of time greater than 30 min [14, 25, 47]. Although narcosis or loss of equilibrium may account for some carp transitioning to an encamped state in the presence of CO_2_, carp might instead be differing in their stress response strategies. Across species, variation in stress response strategies can be large within populations, such as proactive (“fight/flight”) versus reactive (“freeze”) stress responses [22, 26]. For example, juvenile rainbow trout (*Oncorhynchus mykiss*) within a population differ in their stress responses but individuals are consistent in their stress response over time and across contexts [41]. This study aimed to induce escape responses from carp by rapidly increasing CO_2_ concentrations with forced water circulation and contrasts with previous CO_2_ studies where the aim has often been to use CO_2_ as a barrier and induce an avoidance response [12, 25]. The rapid onset of stimulus in our study may have induced differential stress responses strategies of carp and future research would be useful to identify if variation in individual carp responses is consistent over time.

We used Hidden Markov models alongside descriptive Kaplan–Meier estimates to better understand specific experimental effects on the movement of tagged carp. In doing so, we demonstrate a nuanced link between carp movement and latent behaviors in response to experimental variables that would have otherwise gone unnoticed. Based on model estimates, carp movements designated as ‘encamped’ do not imply that the carp were stationary—encamped carp were measured to have some probability of moving, just in a slower and more directed manner. Experimental effects were less pronounced for carp in the encamped behavioral state so that factors that can increase the transition of the carp from an encamped to exploratory behavioral state increased the number and fraction of carp exiting the lock in time. In addition to measuring a negative effect of CO_2_ concentration on carp step lengths and greater transition probabilities from exploratory to an encamped state, we found that mean step length for ‘exploratory’ carp increased with increasing temperature but decreased with increasing temperature for ‘encamped’ carp. With these findings, we were able to obtain a greater understanding of the mechanisms that led to the longer exit times and fractions of carp remaining in the lock during the CO_2_ injection with forced water circulation treatment.

Unlike telemetry studies that only consider detection ability, position accuracy is an important consideration in geospatial telemetry studies. We performed accuracy tests in the lock under the null condition to obtain an estimate of our expected error in carp positions, and obtained, on average, sub-meter accuracy in test tag positions. Given the size of the lock (53 × 11 m) this accuracy was sufficient to track the carp’s movements throughout the lock. However, many of the differences in carp step lengths across the experimental conditions were also measured to be less than 1 m. Despite this, we do not believe this affects our study’s findings and conclusions. First, if positioning error was similar across the experimental conditions, then positioning error would ‘cancel out’ and not affect the differences in step lengths across the experimental conditions. Second, positioning error was, in part, our motivation for quantifying exit times in relation to the HMM results because the carp exit times would be robust against sub-meter positioning error. Given that the HMM results comported well with the Kaplan–Meier estimates of exit times, our conclusions from the HMM are likely valid and unaffected by positioning error. Although it was not feasible to quantify our positioning error for each tag across the experimental conditions, future studies might seek to better estimate position accuracy and error across experimental conditions.

Our approach—descriptive measures combined with HMMs—could help inform implementation strategies of CO_2_ and other behavioral deterrents, such as electric barriers, underwater Acoustic Deterrent Systems (uADS), and bubble screens [8, 15, 33, 53]. For example, a uADS and Bioacoustic Fish Fence (BAFF) have been deployed to reduce upstream movement of invasive carp on the Upper Mississippi and Cumberland Rivers, respectively [8], as well guide juvenile salmon away from dangerous routes in the Sacramento-San Joaquín River Delta [35]. Fine-scale acoustic telemetry data collected across a range of native and invasive fishes during these studies could lend itself to the use of HMM’s to understand fish responses to deterrents and potential variation across individuals. In addition, the use of HMM’s for most deterrent evaluations with acoustic telemetry could provide a standardized approach to compare outcomes across deterrent methods and application scenarios. This apples-to-apples approach could be useful for resource managers to inform decisions on when and where to use each deterrent method and how deterrents might be combined to increase their overall efficacy in limiting the spread of invasive fishes.

Hidden Markov models have rarely been applied to explicitly inform conservation or management strategies. Based on our study, we found that HMMs can be useful in this context for three major reasons. First, when applied in movement ecology HMMs help visualize behavioral dynamics that cannot easily be observed, which is especially important when studying aquatic animals [2, 23, 30]. Second, HMMs can uncover dynamics that are normally unobservable and might be counterintuitive. For example, in one case an HMM approach revealed that while roads can often be barriers to movement for terrestrial carnivores, areas near roads can also simultaneously be preferred for foraging [20]. In this study, our results were contrary to our predictions and brought into focus substantial variation in carp responses to CO_2_ in water—a throughline of decades of research on this topic that can easily be overlooked [43]. Finally, a single application of HMMs can go beyond basic descriptive measures and reveal dynamics that contribute to the “how?” and “why?” of an experimental result, thereby saving time and further research that would normally be needed to gain that valuable insight. Timely access to this type of information is vital to reaching conservation and management goals.

## Conclusions

We describe variation in carp movement responses to the large-scale experimental application of CO_2_ and forced water circulation at a navigation lock. Additionally, we define and discuss the behavioral states and state transitions that contributed to treatment outcomes. We provide evidence that increasing CO_2_ concentrations in water and forced water circulation are effective at expelling common carp from the lock. However, some carp responded to increasing CO_2_ concentrations with forced water circulation by transitioning to an encamped state with shorter step-lengths, resulting in slower exit times and fewer carp leaving the lock than those in the forced water circulation without CO_2_ treatment. Our results may be used to inform the implementation of CO_2_ and/or forced water circulation as a deterrent to fish movement in the future and their combination with other non-physical deterrents. Importantly, our results indicate that acoustic telemetry combined with an HMM approach can be effective at providing fine-scale information about invasive species movement in an experimental context and inform management strategies.

## Supplementary Information

**Additional file 1**.** Supplementary Figure**. Bivariate relationship between the pseudo residuals for step lengths and turning angles (top), and theoretical versus sample quantile plots for turning angle (lower left) and carp step length (bottom right) distributions. Dashed lines represent the 1:1 line of perfect agreement between the theoretical (model) and sample (data) distributions.

## Data Availability

All data used in this study are available at https://doi.org/10.5066/P9B8SRMW [13].

## Abbreviations

BAFF: Bioacoustic Fish Fence
CO_2_: Carbon dioxide
CRW: Correlated random walk
DCP: Digital control panel
HMM: Hidden Markov model
uADS: Underwater Acoustic Deterrent System

## References

[1] Asian Carp Regional Coordinating Committee (ACRCC). “FY2020 Asian Carp Action Plan.” 2020.

[2] Bacheler NM, Michelot T, Cheshire RT, Shertzer KW (2019). Fine-scale movement patterns and behavioral states of gray triggerfish *Balistes capriscus* determined from acoustic telemetry and hidden markov models. Fish Res.

[3] Bernery C, Bellard C, Courchamp F, Brosse S, Gozlan RE, Jarić I, Teletchea F, Leroy B (2022). Freshwater fish invasions: a comprehensive review. Annu Rev Ecol Evol Syst.

[4] Castro-Santos T, Perry R. Time-to-event analysis as a framework for quantifying fish passage performance. In Telemetry Techniques: A User Guide for Fisheries Research, 427–52.: American Fisheries Society, Bethesda, MD. 2012.

[5] California Department of Water Resources. 2012. 2012 Georgiana Slough Non-physical barrier performance evaluation project report. California Department of Water Resources: Sacramento, California. https://data.ca.gov/dataset/2011-and-2012-georgiana-slough-non-physical-barrier-performance-evaluation-gsnpb-11_legacy_repo/resource/a0f2e749-df6b-4690-82ca-ee287750479a

[6] Chapman, Duane C., and Michael H. Hoffman. 2011. “Invasive Asian Carps in North America.” American Fisheries Society Symposium 74. Bethesda, MD: American Fisheries Society.

[7] Conover G, Simmonds R, Whalen M (2007). Management and Control Plan for Bighead, Black, Grass, and Silver Carps in the United States.

[8] Cupp AR, Brey MK, Calfee RD, Chapman DC, Erickson RA, Fischer J, Fritts AK (2021). Emerging control strategies for integrated pest management of invasive carps. J Vert Biol.

[9] Cupp AR, Erickson RA, Fredricks KT, Swyers NM, Hatton TW, Amberg JJ (2017). Responses of invasive silver and bighead carp to a carbon dioxide barrier in outdoor ponds. Can J Fish Aquat Sci.

[10] Cupp AR, Fredricks KT, Porcher ST, Smerud JR, Hartleb CF, Gaikowski MP (2017). Survival and behavioural responses of cool and warm water fish sedated with AQUI-S ® 20E (10% Eugenol) at high loading densities. Aquac Res.

[11] Cupp AR, Lopez AK, Smerud JR, Tix JA, Rivera JM, Swyers NM, Brey MK, Woodley CM, Smith DL, Gaikowski MP (2021). Telemetry evaluation of carbon dioxide as a behavioral deterrent for invasive carps. J Great Lakes Res.

[12] Cupp A, Smerud J, Tix J, Schleis S, Fredricks K, Erickson R, Amberg J (2018). Field evaluation of carbon dioxide as a fish deterrent at a water management structure along the Illinois River. Manag Biol Invas.

[13] Cupp, A.R., Smerud, J.R., Rivera, J.M., Johnson, T.A., Roth, M.F., Nelson, R.G., Raboin, M.J., Jackson, P.R., Soderstrom, C.M., Duncker, J.J., Reneau, P.C., Owens, D.W., Plumb, J.M., Sholtis, M., Swyers, N., Hatton, T., Smith, D., and Suski, C., 2023, Acoustic Telemetry Evaluation of Invasive Carp in Kaukauna, Wisconsin (Summer 2019): U.S. Geological Survey data release, 10.5066/P9B8SRMW.

[14] Dennis CE, Adhikari S, Wright AW, Suski CD (2016). Molecular, behavioral, and performance responses of Juvenile Largemouth Bass acclimated to an elevated carbon dioxide environment. J Comp Physiol B.

[15] Dennis CE, Zielinski D, Sorensen PW (2019). A complex sound coupled with an air curtain blocks invasive carp passage without habituation in a laboratory flume. Biol Invas.

[16] Donaldson MR, Amberg J, Adhikari S, Cupp A, Jensen N, Romine J, Wright A, Gaikowski M, Suski CD (2016). Carbon dioxide as a tool to deter the movement of invasive bigheaded carps. Trans Am Fish Soc.

[17] Ehrenberg JE, Steig TW (2003). Improved techniques for studying the temporal and spatial behavior of fish in a fixed location. ICES J Marine Sci.

[18] Ehrenberg JE, Steig TW (2009). A study of the relationship between tag-signal characteristics and achievable performances in acoustic fish-tag studies. ICES J Marine Sci.

[19] Enders EC, Boisclair D, Roy AG (2005). A model of total swimming costs in turbulent flow for Juvenile Atlantic Salmon (*Salmo salar*). Can J Fish Aqua Sci.

[20] Ferreira EM, Valerio F, Medinas D, Fernandes N, Craveiro J, Costa P, Silva JP, Carrapato C, Mira A, Santos SM (2022). Assessing behaviour states of a forest carnivore in a road-dominated landscape using hidden markov models. Nature Conserv.

[21] Gallardo B, Clavero M, Sánchez MI, Vilà M (2016). Global ecological impacts of invasive species in aquatic ecosystems. Glob Change Biol.

[22] Hasler CT, Bouyoucos IA, Suski CD (2017). Tolerance to hypercarbia is repeatable and related to a component of the metabolic phenotype in a freshwater fish. Physiol Biochem Zool.

[23] Holt MM, Tennessen JB, Ward EJ, Bradley Hanson M, Emmons CK, Giles DA, Hogan JT (2021). Effects of vessel distance and sex on the behavior of endangered killer whales. Front Mar Sci.

[24] Johnson DC. Fit continuous-time correlated random walk models to animal movement data. R software package. 2020.

[25] Kates D, Dennis C, Noatch MR, Suski CD (2012). “Responses of Native and Invasive Fishes to Carbon Dioxide: Potential for a Nonphysical Barrier to Fish Dispersal.” Edited by D. L. MacLatchy. Can J Fish Aquatic Sci.

[26] Koolhaas JM, Korte SM, De Boer SF, Van Der Vegt BJ, Van Reenen CG, Hopster H, De Jong IC, Ruis MAW, Blokhuis HJ (1999). Coping styles in animals: current status in behavior and stress-physiology. Neurosci Biobehav Rev.

[27] Langrock R, King R, Matthiopoulos J, Thomas L, Fortin D, Morales JM (2012). Flexible and practical modeling of animal telemetry data: hidden Markov models and extensions. Ecology.

[28] Leos-Barajas V, Photopoulou T, Langrock R, Patterson TA, Watanabe YY, Murgatroyd M, Papastamatiou YP (2017). “Analysis of Animal Accelerometer Data Using Hidden Markov Models.” Edited by Robert B. O’Hara. Methods Ecol Evol.

[29] Maes J, Turnpenny AWH, Lambert DR, Nedwell JR, Parmentier A, Ollevier F (2004). Field evaluation of a sound system to reduce estuarine fish intake rates at a power plant cooling water inlet. J Fish Biol.

[30] McClintock BT, Langrock R, Gimenez O, Cam E, Borchers DL, Glennie R, Patterson TA (2020). Uncovering ecological state dynamics with hidden Markov models. Edited by Tim Coulson. Ecol Lett.

[31] McClintock BT, Michelot T (2018). “MomentuHMM: R Package for Generalized Hidden Markov Models of Animal Movement.” Edited by Sarah Goslee. Methods Ecol Evol.

[32] Nathan R, Getz WM, Revilla E, Holyoak M, Kadmon R, Saltz D, Smouse PE (2008). A movement ecology paradigm for unifying organismal movement research. Proc Natl Acad Sci.

[33] Noatch MR, Suski CD (2012). Non-physical barriers to deter fish movements. Environ Rev.

[34] Parker AD, Glover DC, Finney ST, Bradley Rogers P, Stewart JG, Simmonds RL (2016). “Fish Distribution, Abundance, and Behavioral Interactions within a Large Electric Dispersal Barrier Designed to Prevent Asian Carp Movement”. Edited by Jordan Rosenfeld. Can J Fish Aquat Sci.

[35] Perry RW, Romine JG, Adams NS, Blake AR, Burau JR, Johnston SV, Liedtke TL (2014). Using a non-physical behavioral barrier to alter migration routing of juvenile Chinook salmon in the Sacramento-San Joaquin River Delta. River Res Appl.

[36] Pohle J, Langrock R, van Beest FM, Schmidt NM (2017). Selecting the number of states in hidden markov models: pragmatic solutions illustrated using animal movement. J Agric Biol Environ Stat.

[37] Prada AF, George AE, Stahlschmidt BH, Ryan Jackson P, Chapman DC, Tinoco RO (2021). Using turbulence to identify preferential areas for grass carp (*Ctenopharyngodon Idella* ) larvae in streams: a laboratory study. Water Resour Res.

[38] Prada AF, George AE, Stahlschmidt BH, Jackson PR, Chapman DC, Tinoco RO (2020). Influence of turbulence and in-stream structures on the transport and survival of grass carp eggs and larvae at various developmental stages. Aquat Sci.

[39] R Core Team. “R: A Language and Environment for Statistical Computing.” Vienna, Austria: R Foundation for Statistical Computing. 2020. https://www.R-project.org/.

[40] Romine JG, Jensen NR, Parsley MJ, Gaugush RF, Severson TJ, Hatton TW, Adams RF, Gaikowski MP (2015). Response of bighead carp and silver carp to repeated water gun operation in an enclosed shallow pond. North Am J Fish Manag.

[41] Schjolden J, Stoskhus A, Winberg S (2005). Does individual variation in stress responses and agonistic behavior reflect divergent stress coping strategies in Juvenile Rainbow Trout?. Physiol Biochem Zool.

[42] Silva AT, Katopodis C, Santos JM, Ferreira MT, Pinheiro AN (2012). Cyprinid swimming behaviour in response to turbulent flow. Ecol Eng.

[43] Suski CD (2020). Development of carbon dioxide barriers to deter invasive fishes: insights and lessons learned from bigheaded carp. Fishes.

[44] Tan J, Gao Z, Dai H, Yang Z, Shi X (2019). Effects of turbulence and velocity on the movement behaviour of bighead carp (*Hypophthalmichthys nobilis*) in an experimental vertical slot fishway. Ecol Eng.

[45] Tarrade L, Pineau G, Calluaud D, Texier A, David L, Larinier M (2011). Detailed experimental study of hydrodynamic turbulent flows generated in vertical slot fishways. Environ Fluid Mech.

[46] Tessema A, Getahun A, Mengistou S, Fetahi T, Dejen E (2020). Reproductive Biology of Common Carp (Cyprinus Carpio Linnaeus, 1758) in Lake Hayq, Ethiopia. Fisheries and Aquatic Sciences.

[47] Tix JA, Cupp AR, Smerud JR, Erickson RA, Fredricks KT, Amberg JJ, Suski CD (2018). Temperature dependent effects of carbon dioxide on avoidance behaviors in bigheaded carps. Biol Invas.

[48] Tucker EK, Suski CD (2019). Presence of conspecifics reduces between-individual variation and increases avoidance of multiple stressors in Bluegill. Anim Behav.

[49] Tucker EK, Suski CD, Philipp MA, Jeffrey JD, Hasler CT (2019). Glucocorticoid and behavioral variation in relation to carbon dioxide avoidance across two experiments in freshwater teleost fishes. Biol Invasions.

[50] USEPA. “Carbon Dioxide-Carp: EPA Registration Number 6704–95.” Federal Insecticide, Fungicide and Rodenticide Act. USEPA. 2019. https://www3.epa.gov/pesticides/chem_search/ppls/006704-00095-20190419.pdf.

[51] Vetter BJ, Cupp AR, Fredricks KT, Gaikowski MP, Mensinger AF (2015). Acoustical deterrence of silver carp (*Hypophthalmichthys molitrix*). Biol Invasions.

[52] Vetter BJ, Murchy KA, Cupp AR, Amberg JJ, Gaikowski MP, Mensinger AF (2017). Acoustic deterrence of bighead carp (*Hypophthalmichthys nobilis*) to a broadband sound stimulus. J Great Lakes Res.

[53] Zielinski DP, Sorensen PW (2016). Bubble curtain deflection screen diverts the movement of both Asian and Common Carp. North Am J Fish Manag.

[54] Zolper TJ, Cupp AR, Smith DL (2019). Investigating the mixing efficiencies of liquid-to-liquid chemical injection manifolds for aquatic invasive species management. J Fluids Eng.

[55] Zolper TJ, Smith DL, Ryan Jackson P, Cupp AR (2022). Performance of a Carbon Dioxide Injection System at a Navigation Lock to Control the Spread of Aquatic Invasive Species. J Environ Eng.

