# Supplementary material for: Movement and behavioral states of common carp (Cyprinus carpio) in response to a behavioral deterrent in a navigational lock
Source: Mov Ecol. 2023 Jul 26;11:42. doi: 10.1186/s40462-023-00396-z (PMC10373248; doi:10.1186/s40462-023-00396-z)
Supplement: Supplementary file 1 — Additional file 1. Supplementary Figure. Bivariate relationship between the pseudo residuals for step lengths and turning angles (top), and theoretical versus sample quantile plots for turning angle (lower left) and carp step length (bottom right) distributions. Dashed lines represent the 1:1 line of perfect agreement between the theoretical (model) and sample (data) distributions. [file 40462_2023_396_MOESM1_ESM.docx]

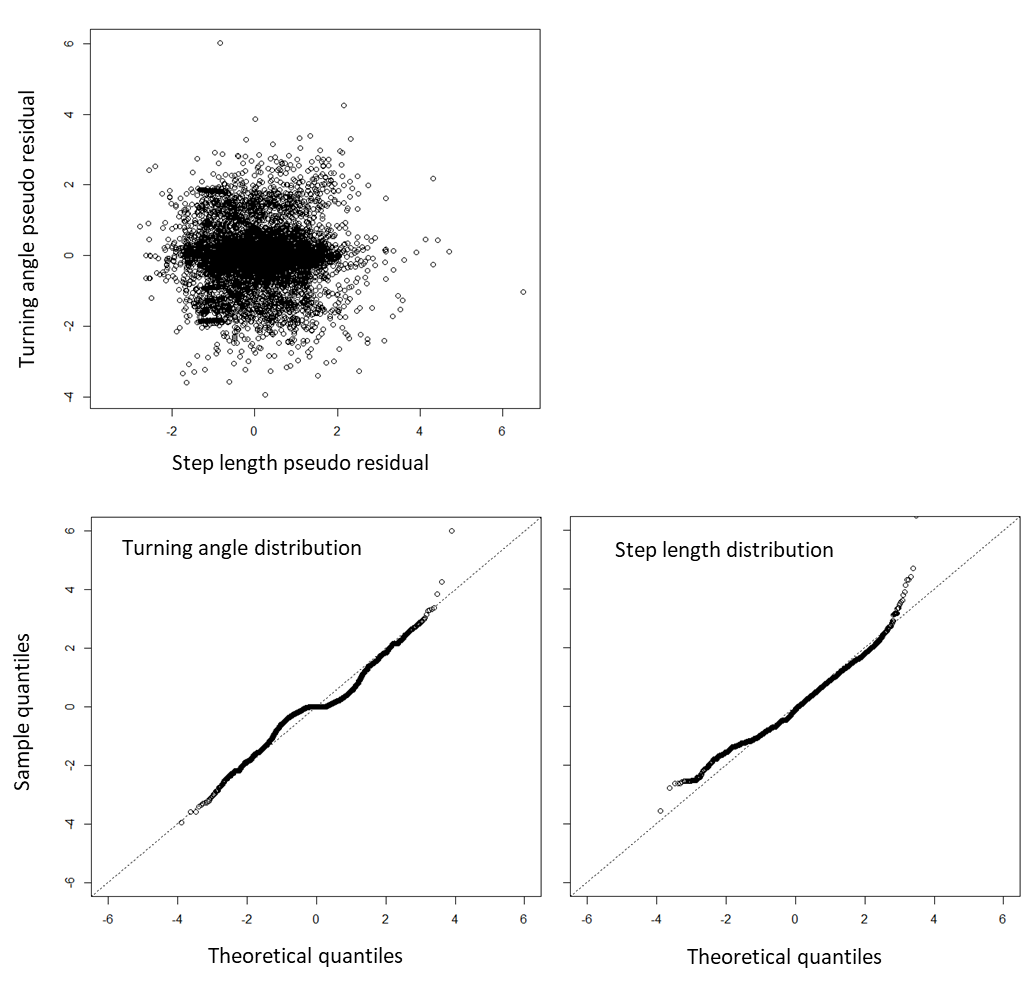


Supplementary Figure. –  Bivariate relationship between the pseudo residuals for step lengths and turning angles (top), and theoretical versus sample quantile plots for turning angle (lower left) and carp step length (bottom right) distributions. Dashed lines represent the 1:1 line of perfect agreement between the theoretical (model) and sample (data) distributions.
